# Supplementary material for: OH-EpiCap: a semi-quantitative tool for the evaluation of One Health epidemiological surveillance capacities and capabilities
Source: Front Public Health. 2023 May 11;11:1053986. doi: 10.3389/fpubh.2023.1053986 (PMC10213933; doi:10.3389/fpubh.2023.1053986)
Supplement: Supplementary file 2 [file Data_Sheet_2.pdf]

Supplementary file S2. Example of a OH-EpiCap report generated by the web application, displaying graphical representations of the evaluation results at the dimension, target and indicator levels.

OH-EpiCap Index and Dimensions

OH-EpiCap and Dimension indices represent mean scores over all questions, expressed as a percentage.

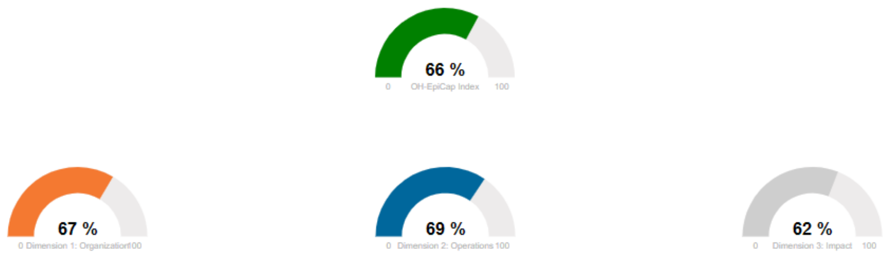

Targets

This section shows the results of the twelve targets, divided across the three dimensions.
Scores range 1-4, with higher values suggesting better adherence to the One Health principle (better integration of sectors), and lower values suggesting improvements may be beneficial.
Users are encouraged to hover over data points to view a breakdown of each target score.

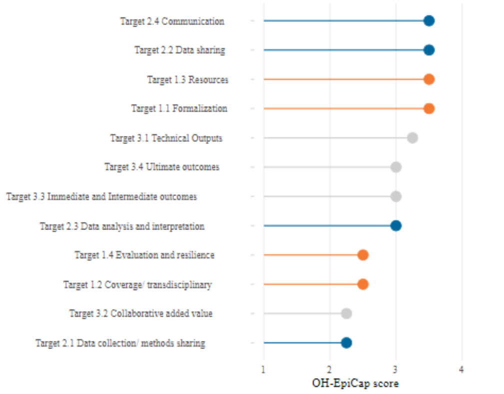

Download Plot (SVG)

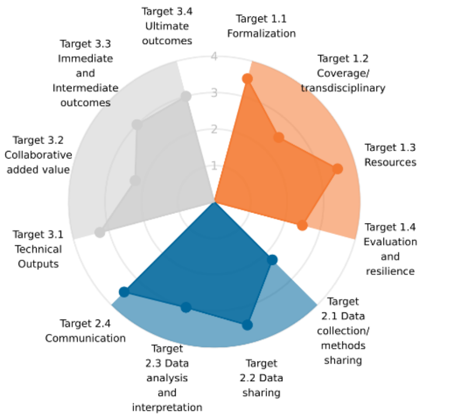

Download Plot (SVG)

Targets demonstrating **good adherence** to One Health principles are: **Target 1.1 Formalization, Target 1.3 Resources, Target 2.2 Data sharing, Target 2.4 Communication, Target 3.1 Technical Outputs**.
Targets that would **most benefit from improvement** are: **None**.

Dimension 1: Organization

This section shows the results across all indicators within the four targets of Dimension 1 (Organization).

Scores range 1-4, with higher values suggesting better adherence to the One Health principle (better integration of sectors), and lower values suggesting improvements may be beneficial.

Indicators labelled in grey indicate a question was answered with NA. Users are encouraged to hover over plotted data points to view the wording of the chosen indicator level, and any comments that may have been added in connection with a particular question.

Indicators demonstrating **good adherence** to One Health principles are: **Common aim, Supporting documentations, Disciplines, Human resources, Shared ressources, Training, Internal evaluation**.

Indicators that would **most benefit from improvement** are: **Sectors, External evaluation**.

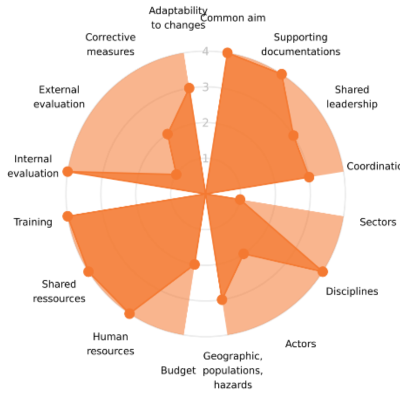

Download Plot (SVG)

Dimension 2: Operations

This section shows the results across all indicators within the four targets of Dimension 2 (Operations).

Scores range 1-4, with higher values suggesting better adherence to the One Health principle (better integration of sectors), and lower values suggesting improvements may be beneficial.

Indicators labelled in grey indicate a question was answered with NA. Users are encouraged to hover over plotted data points to view the wording of the chosen indicator level, and any comments that may have been added in connection with a particular question.

Indicators demonstrating **good adherence** to One Health principles are: **Data collection, Data quality, FAIR data, Sharing techniques, Sharing expertise, Internal communication, External communication, Dissemination**.

Indicators that would **most benefit from improvement** are: **Data warehouse, Joint analysis**.

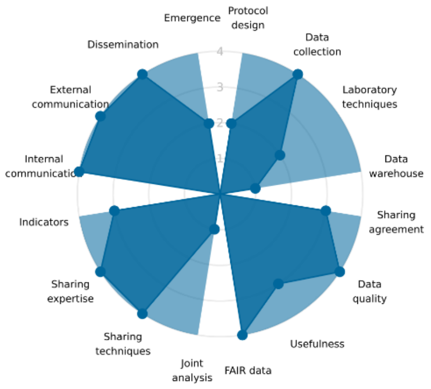

Download Plot (SVG)

Dimension 3: Impact

This section shows the results across all indicators within the four targets of Dimension 3 (Impact).

Scores range 1-4, with higher values suggesting better adherence to the One Health principle (better integration of sectors), and lower values suggesting improvements may be beneficial.

Indicators labelled in grey indicate a question was answered with NA. Users are encouraged to hover over plotted data points to view the wording of the chosen indicator level, and any comments that may have been added in connection with a particular question.

Indicators demonstrating **good adherence** to One Health principles are: **Effectiveness, Advocacy, Policy changes, Behavioral changes**.

Indicators that would **most benefit from improvement** are: **OH team**.

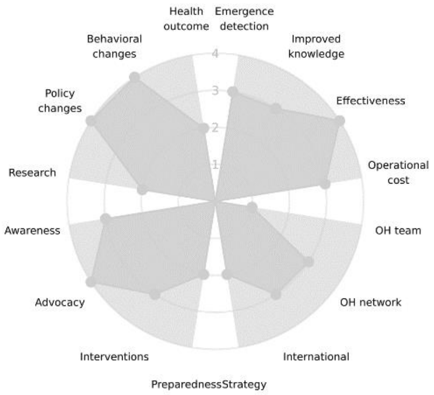

Download Plot (SVG)
